# Supplementary figures and images for: Regulation of store-operated Ca2+ entry by IP3 receptors independent of their ability to release Ca2+
Source: eLife. 2023 Jul 19;12:e80447. doi: 10.7554/eLife.80447 (PMC10406432; doi:10.7554/eLife.80447)

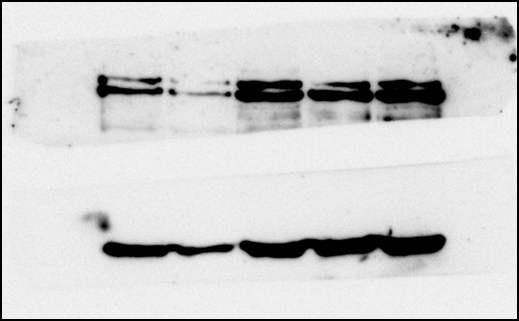

Supplement: Figure 1—source data 1. [file elife-80447-fig1-data1.zip › Figure 1 source data/Blot image_Figure1B.tif]

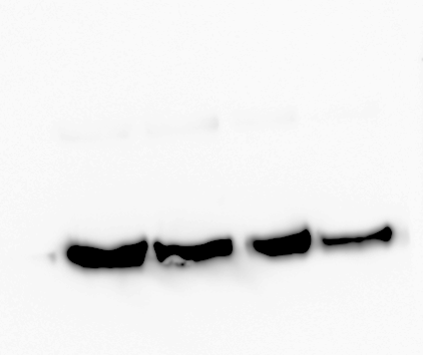

Supplement: Figure 1—figure supplement 1—source data 1. [file elife-80447-fig1-figsupp1-data1.zip › Figure 1- figure supplement 1 source data/Blot image 1_G.tif]

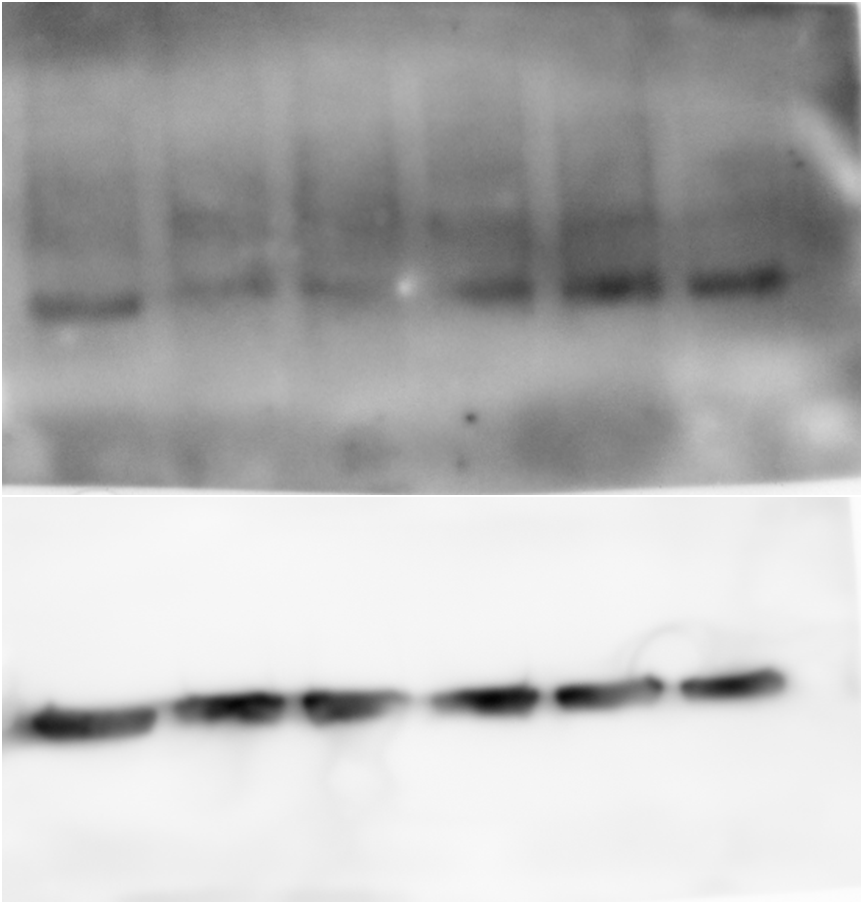

Supplement: Figure 1—figure supplement 1—source data 1. [file elife-80447-fig1-figsupp1-data1.zip › Figure 1- figure supplement 1 source data/Blot image 2_G.tif]

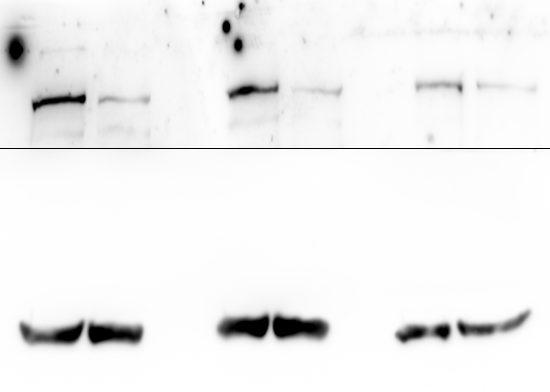

Supplement: Figure 2—source data 1. [file elife-80447-fig2-data1.zip › Figure 2 source data/Blot image 1_Figure 2A.tif]

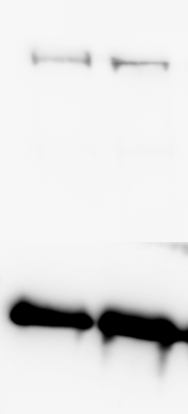

Supplement: Figure 2—source data 1. [file elife-80447-fig2-data1.zip › Figure 2 source data/Blot image 2_Figure 2A.tif]

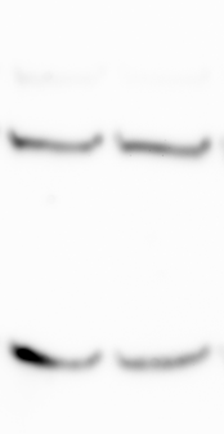

Supplement: Figure 2—figure supplement 1—source data 1. [file elife-80447-fig2-figsupp1-data1.zip › Figure 2- figure supplement 1 source data/Blot image 1_E.tif]

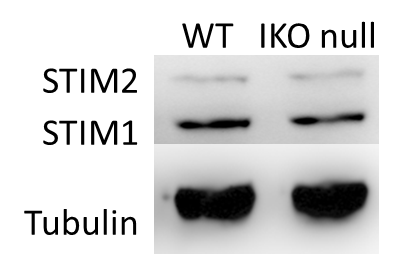

Supplement: Figure 2—figure supplement 1—source data 1. [file elife-80447-fig2-figsupp1-data1.zip › Figure 2- figure supplement 1 source data/BLot image 1_G.tif]

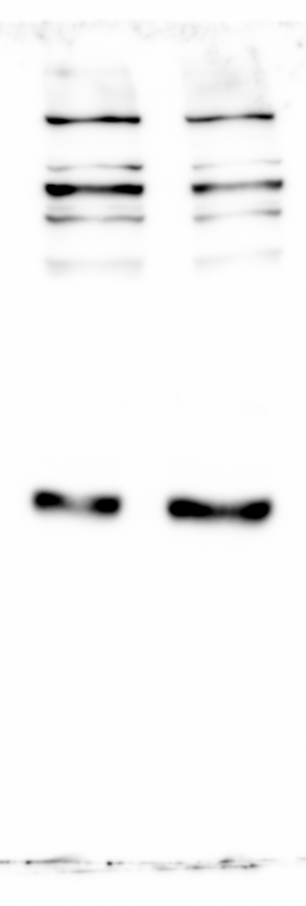

Supplement: Figure 2—figure supplement 1—source data 1. [file elife-80447-fig2-figsupp1-data1.zip › Figure 2- figure supplement 1 source data/Blot image 1_M.tif]

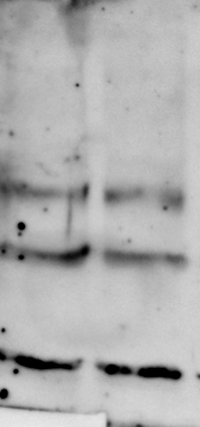

Supplement: Figure 2—figure supplement 1—source data 1. [file elife-80447-fig2-figsupp1-data1.zip › Figure 2- figure supplement 1 source data/Blot image 2_E.tif]

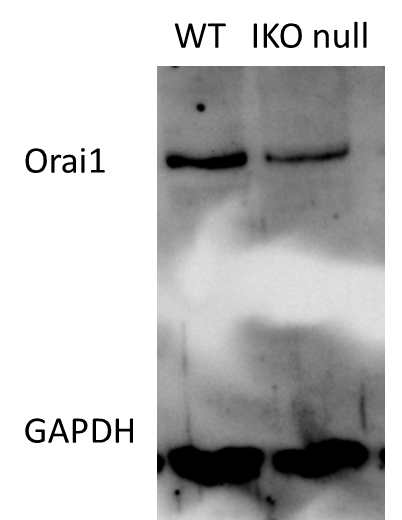

Supplement: Figure 2—figure supplement 1—source data 1. [file elife-80447-fig2-figsupp1-data1.zip › Figure 2- figure supplement 1 source data/BLot image 2_G.tif]

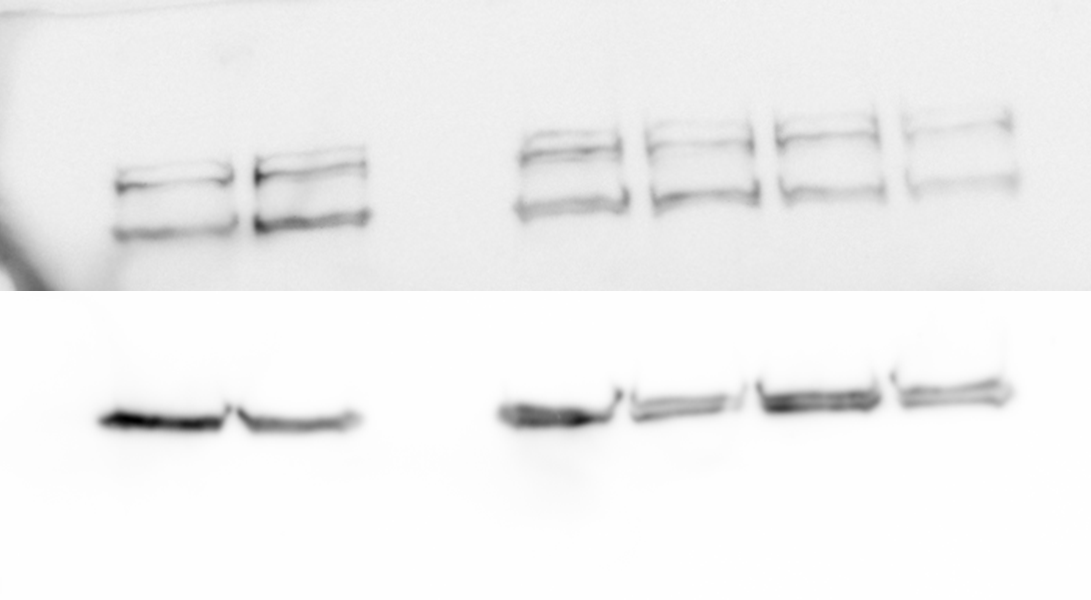

Supplement: Figure 2—figure supplement 3—source data 1. [file elife-80447-fig2-figsupp3-data1.zip › Figure 2- figure supplement 3 source data/Blot image 1 _A.tif]

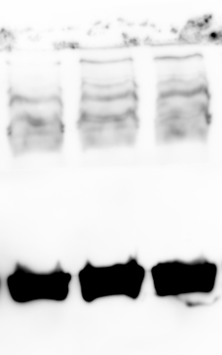

Supplement: Figure 3—figure supplement 1—source data 1. [file elife-80447-fig3-figsupp1-data1.zip › Figure 3- figure supplement 1 source data/Blot image 1_A.tif]

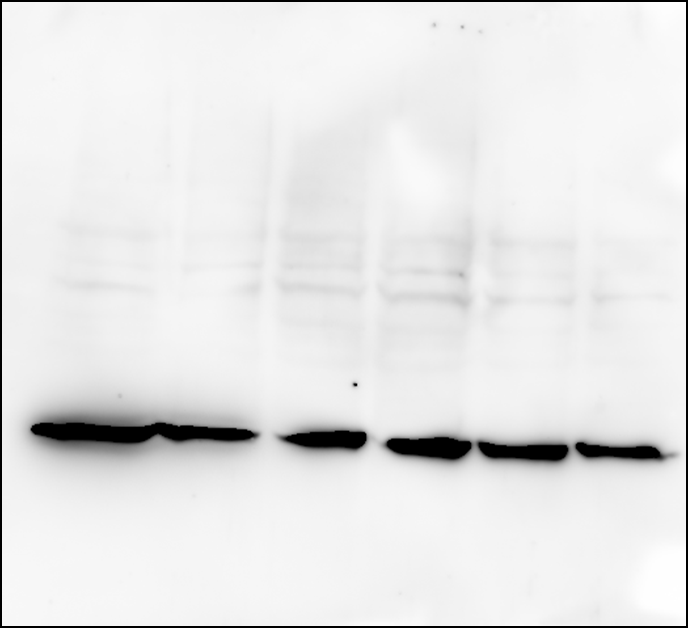

Supplement: Figure 3—figure supplement 1—source data 1. [file elife-80447-fig3-figsupp1-data1.zip › Figure 3- figure supplement 1 source data/Blot image 1_F.tif]

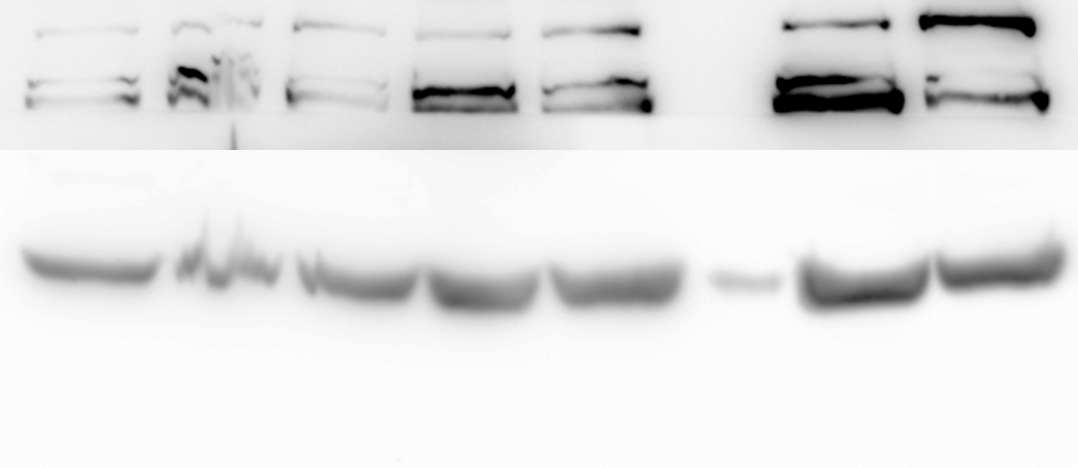

Supplement: Figure 3—figure supplement 1—source data 1. [file elife-80447-fig3-figsupp1-data1.zip › Figure 3- figure supplement 1 source data/Blot image 1_I.tif]
