# Supplementary material for: Regulation of store-operated Ca2+ entry by IP3 receptors independent of their ability to release Ca2+
Source: eLife. 2023 Jul 19;12:e80447. doi: 10.7554/eLife.80447 (PMC10406432; doi:10.7554/eLife.80447)
Supplement: Figure 1—figure supplement 1—source data 1. [file elife-80447-fig1-figsupp1-data1.zip › Figure 1- figure supplement 1 source data/Figure 1- figure supplement 1- data 2.pdf]

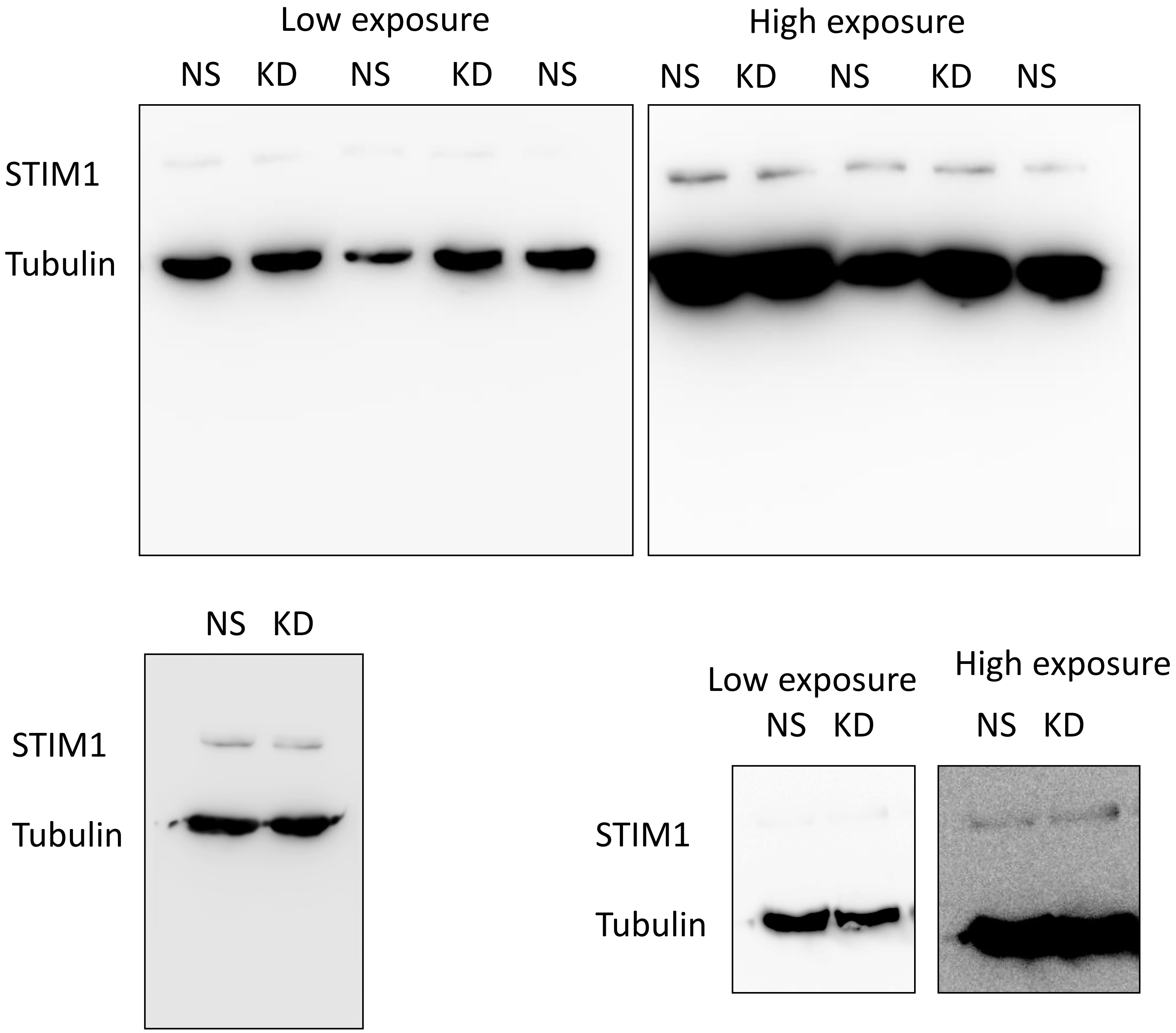

NS- Control shRNA (shRNA NS), KD- IP_3R1 shRNA

In 1st and 3rd blot I had to use differenet exposure time for STIM1 and Tubulin

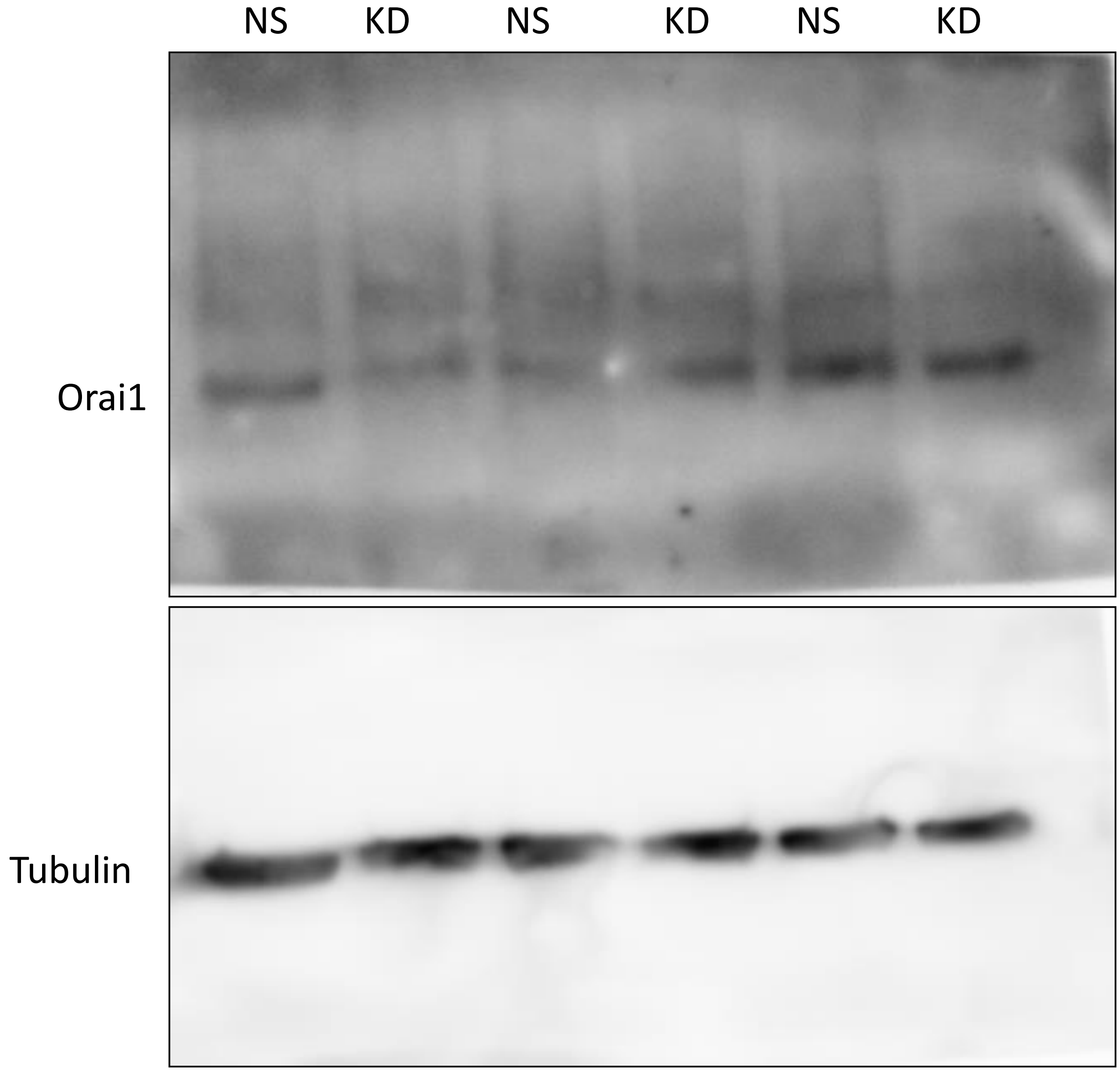

NS- Control shRNA (shRNA NS), KD-IP$_3$R1 shRNA, Each NS or KD indicates each biological replicates.
