# Supplementary material for: Regulation of store-operated Ca2+ entry by IP3 receptors independent of their ability to release Ca2+
Source: eLife. 2023 Jul 19;12:e80447. doi: 10.7554/eLife.80447 (PMC10406432; doi:10.7554/eLife.80447)
Supplement: Figure 2—figure supplement 1—source data 1. [file elife-80447-fig2-figsupp1-data1.zip › Figure 2- figure supplement 1 source data/Figure 2- figure supplement 1- data 2.pdf]

STIM1 Western Blot (Panel E)

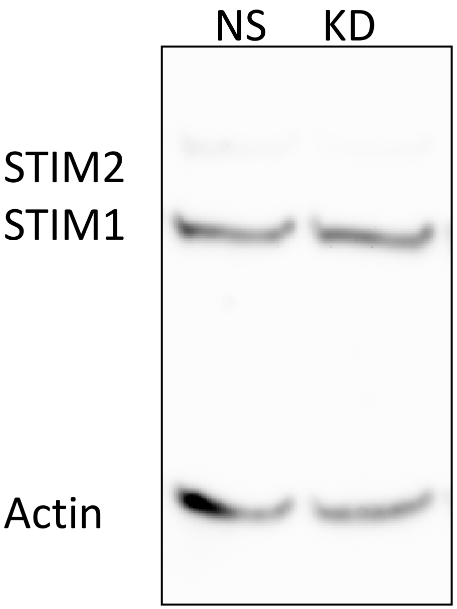

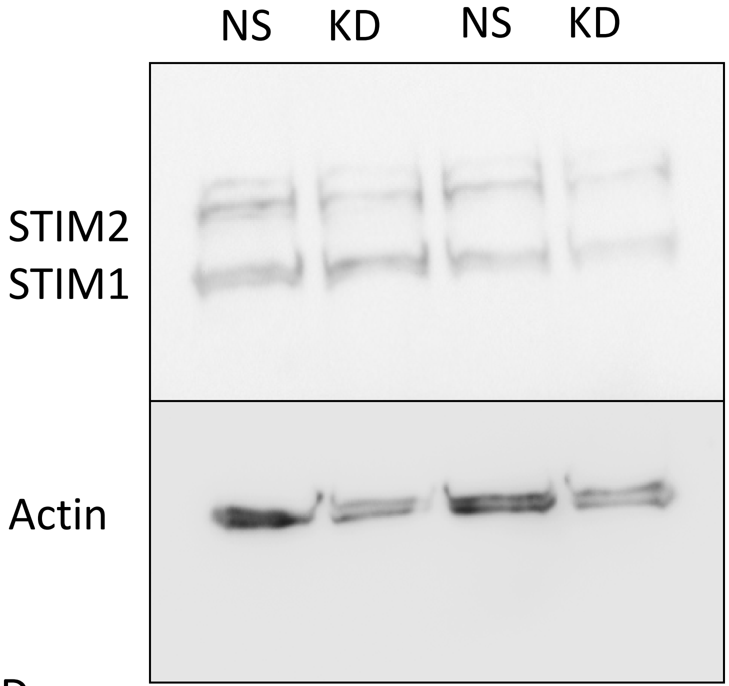

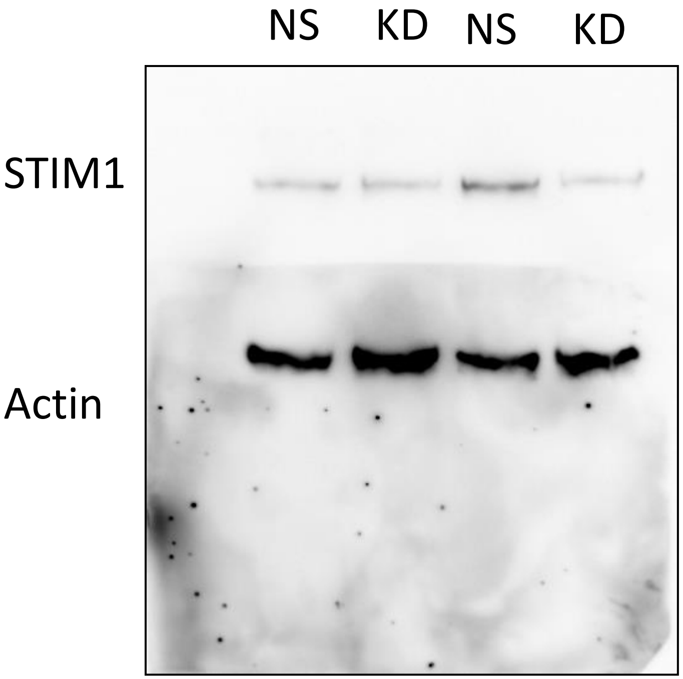

Orai1 Western blot(Panel E)

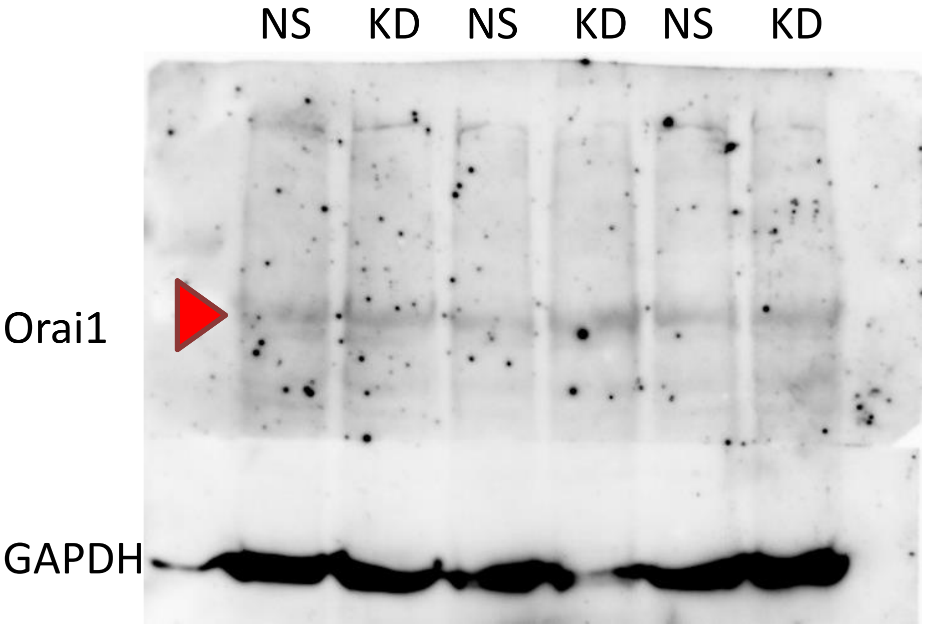

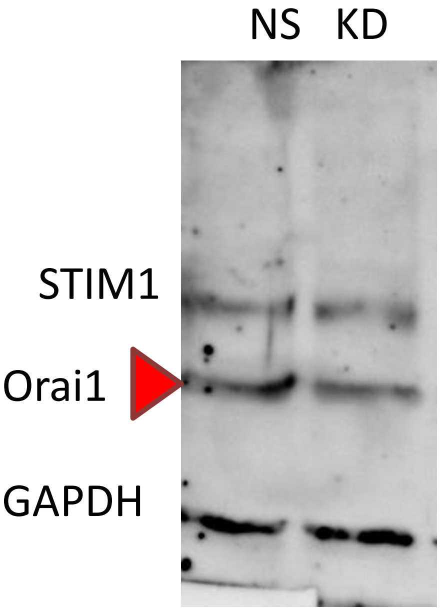

IP$_3$R1 Western blot(Panel M)

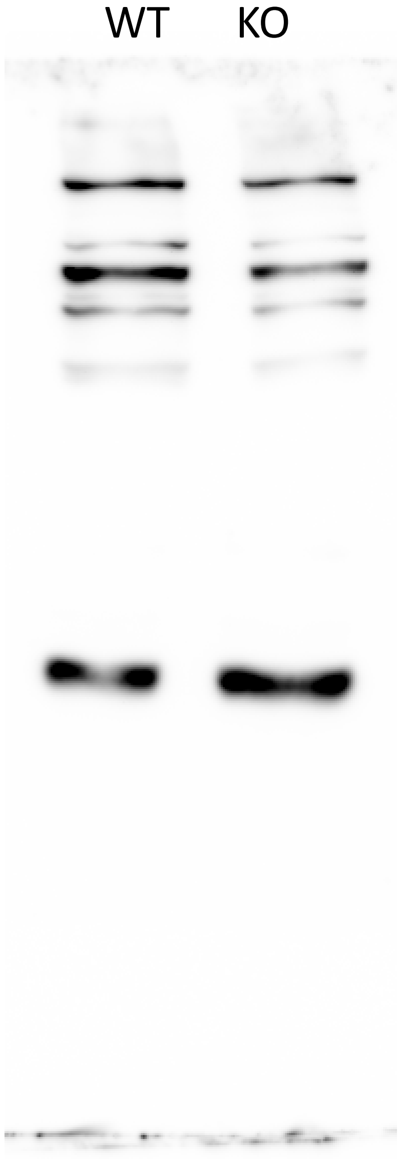

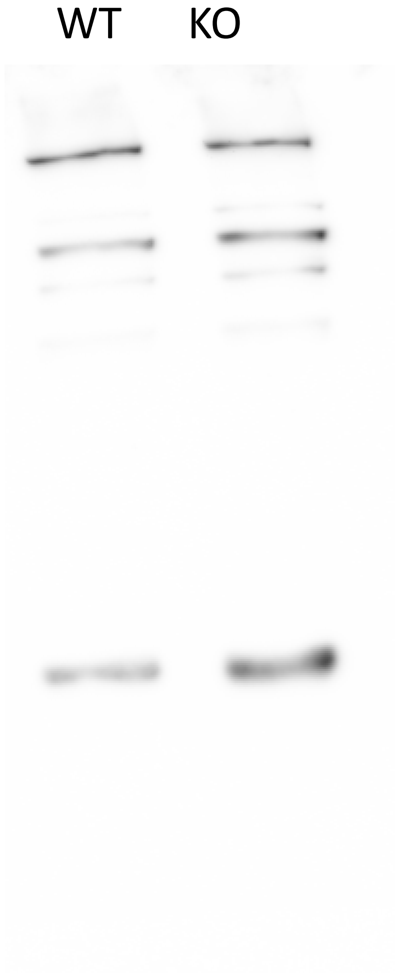

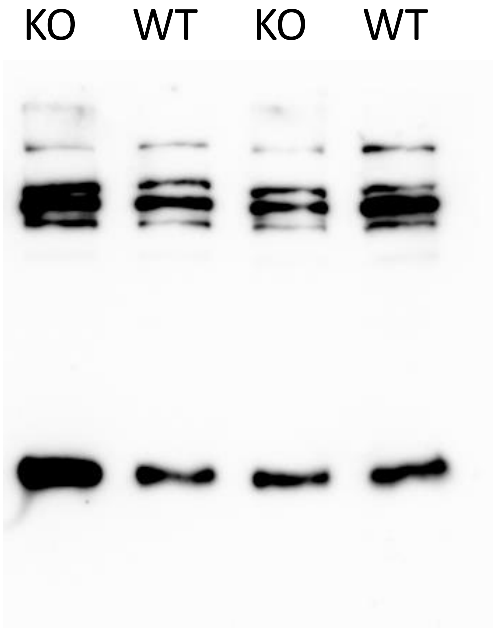

WT-Wild type SH-SY5Y
KO- CRISPR Cas9n edited SH-SY5Y
Each WT/KO indicates each biological replicates
