# Supplementary material for: Regulation of store-operated Ca2+ entry by IP3 receptors independent of their ability to release Ca2+
Source: eLife. 2023 Jul 19;12:e80447. doi: 10.7554/eLife.80447 (PMC10406432; doi:10.7554/eLife.80447)
Supplement: Figure 2—figure supplement 3—source data 1. [file elife-80447-fig2-figsupp3-data1.zip › Figure 2- figure supplement 3 source data/Figure 2- figure supplement 3 source data 2.pdf]

IP$_3$R1 Western blot in HEK cells

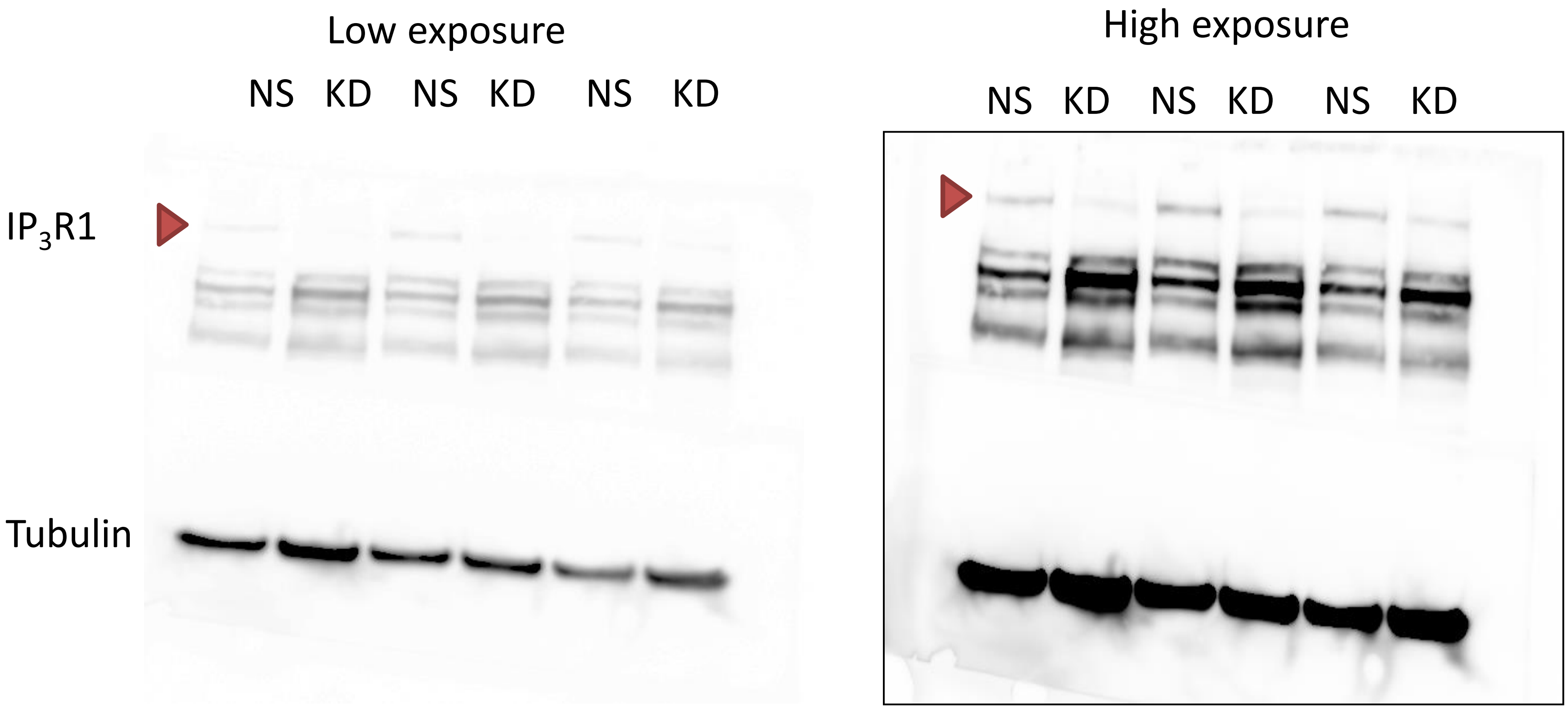

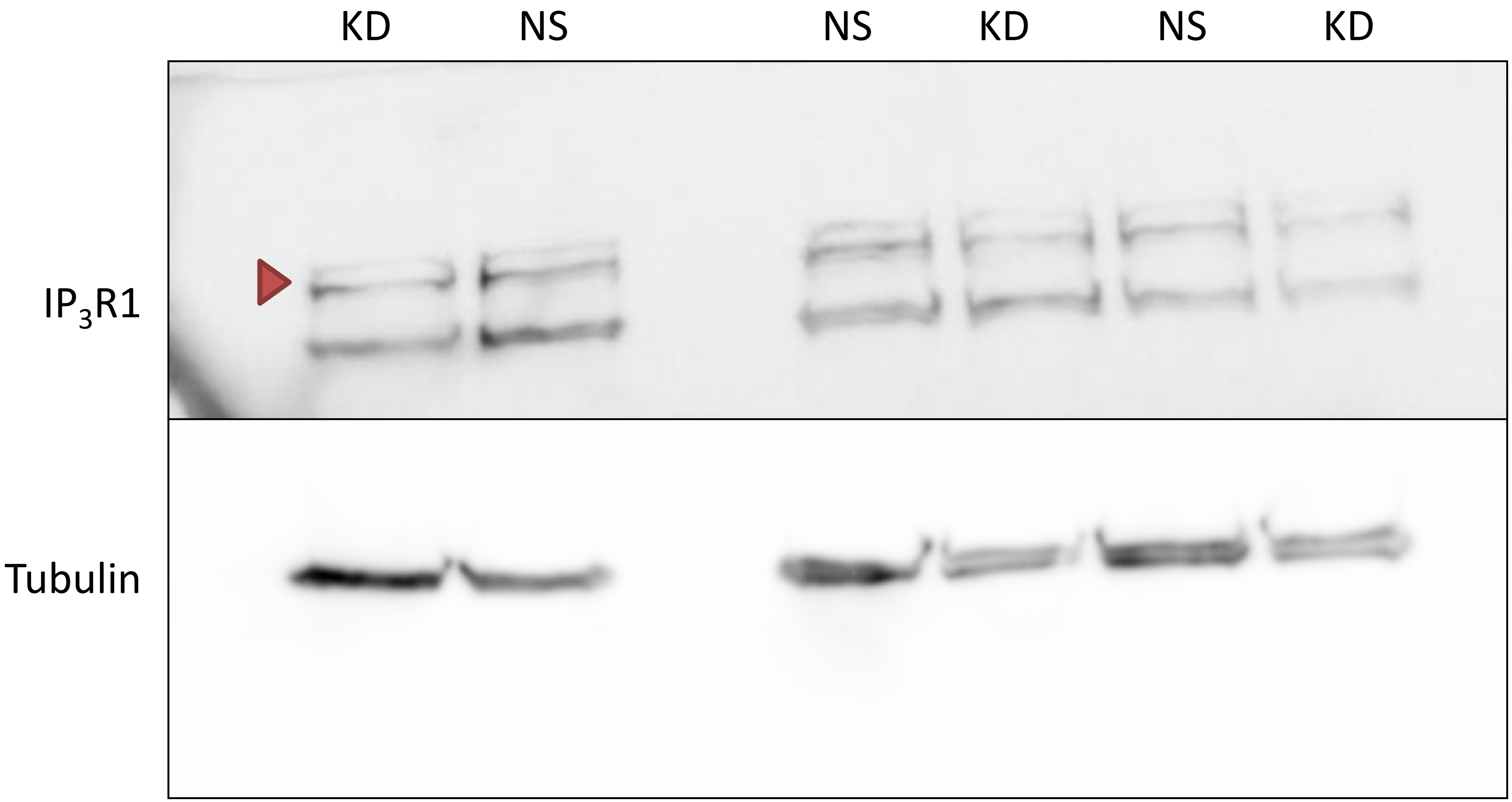

NS- control shRNA (NS shRNA)
KD- IP_3R1 shRNA
Blots were cut before primary antibody incubation to inhibit cross reactivity.
Every NS and KD indicates individual replicates.
