# Supplementary material for: Regulation of store-operated Ca2+ entry by IP3 receptors independent of their ability to release Ca2+
Source: eLife. 2023 Jul 19;12:e80447. doi: 10.7554/eLife.80447 (PMC10406432; doi:10.7554/eLife.80447)
Supplement: Figure 3—figure supplement 1—source data 1. [file elife-80447-fig3-figsupp1-data1.zip › Figure 3- figure supplement 1 source data/Figure 3- figure supplement 1_source data 2.pdf]

IP_3R1 Western blotting (Panel A and B)

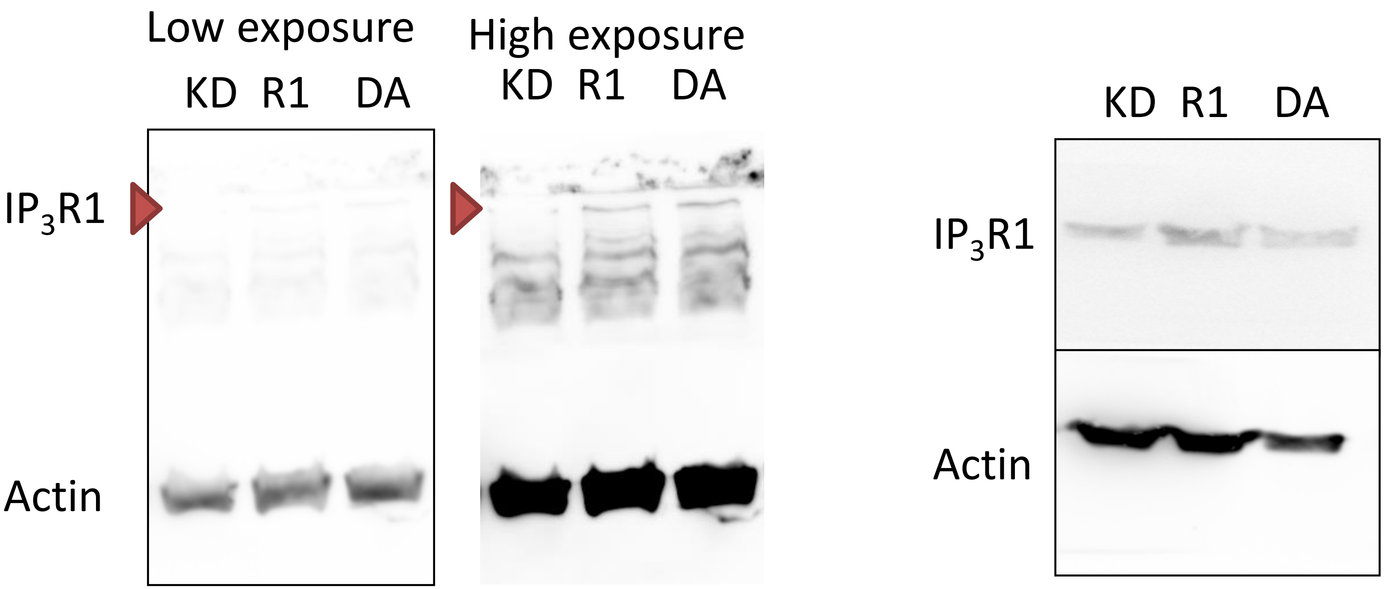

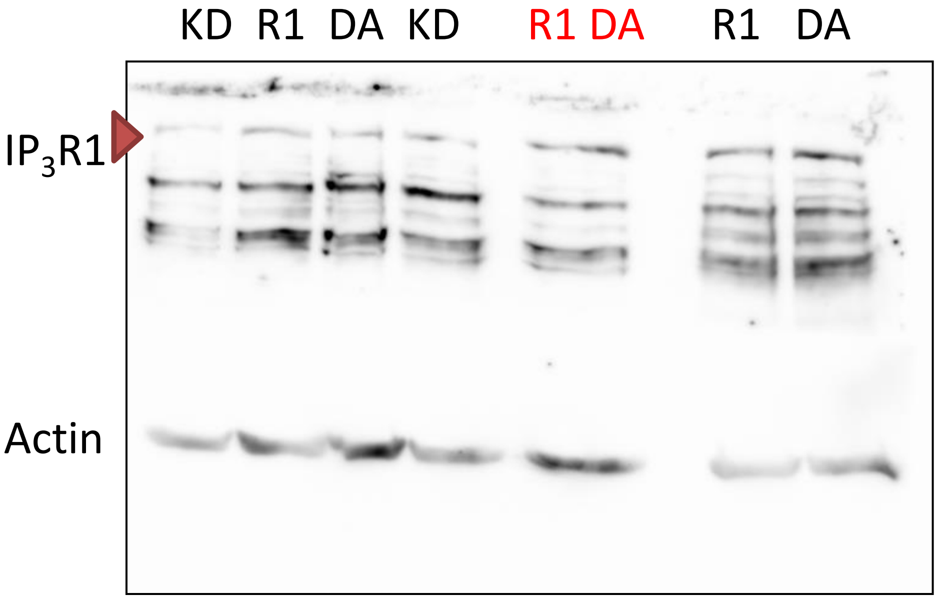

KD-IP_3R1 shRNA
R1-IP_3R1 shRNA+IP_3R1
DA-IP_3R1 shRNA+IP_3R1^{DA}

Each KD/R1/DA indicates individual biological replicates

Red labeled (RA DA) lanes were excluded as the gel lanes were merged

Orai1 Western blotting (Panel F and G)

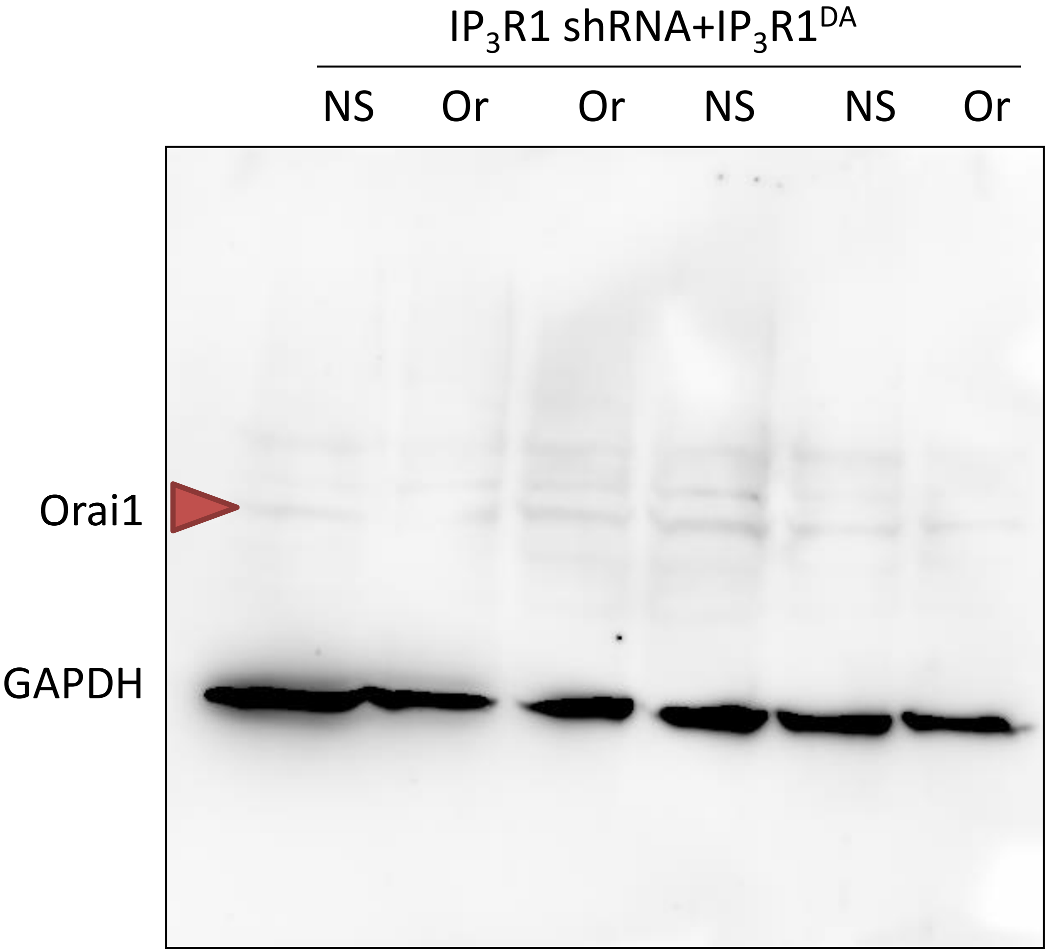

NS- siControl
Or- siOrai1
Each NS/Or indicates individual biological replicates

IP_3R1 Western blotting (Panel I)

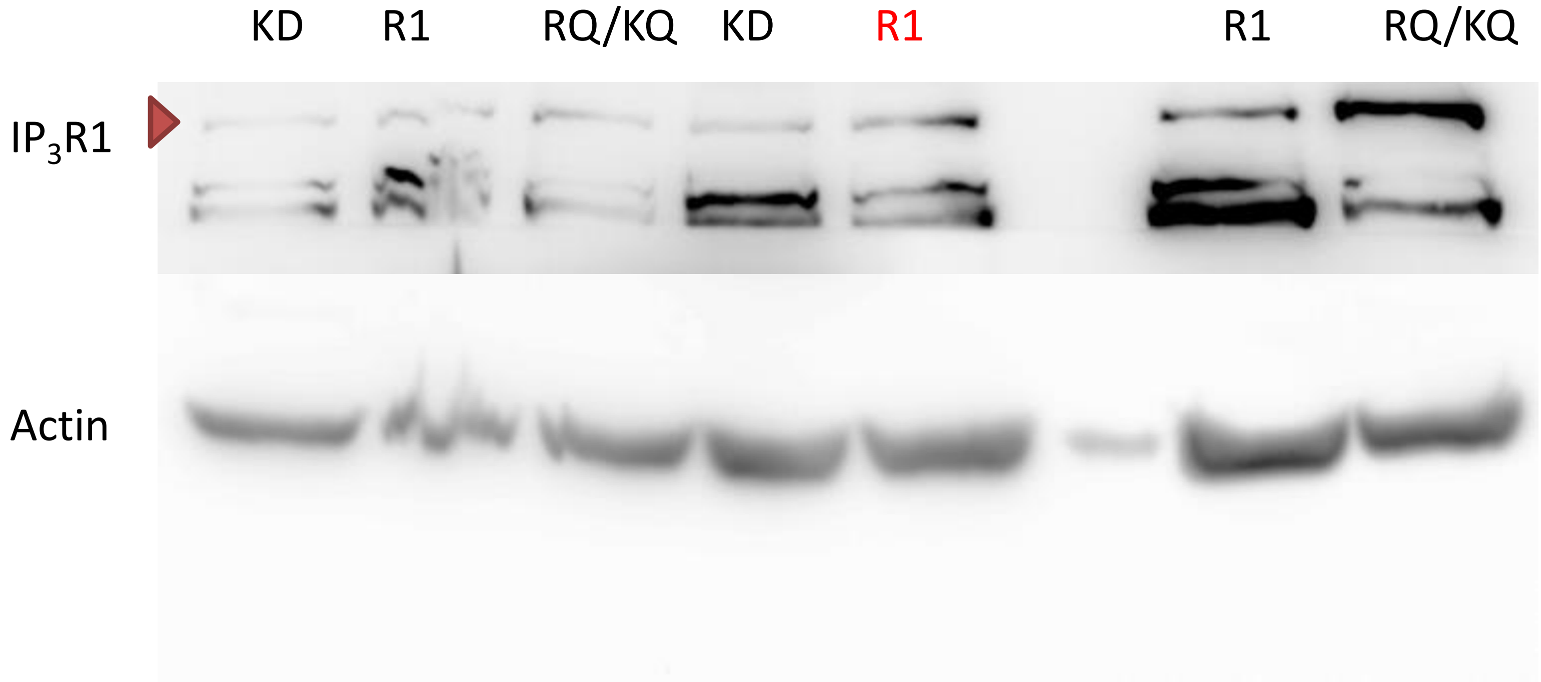

KD-IP_3R1 shRNA
R1-IP_3R1 shRNA+IP_3R1
RQ/KQ-IP_3R1 shRNA+$IP_3R1^{RQ/KQ}$
Each NS/R1 or RQ/KQ indicates each biological replicates

Red labeled (RA) lane was excluded as the gel lane was leaked
