# Supplementary material for: Regulation of store-operated Ca2+ entry by IP3 receptors independent of their ability to release Ca2+
Source: eLife. 2023 Jul 19;12:e80447. doi: 10.7554/eLife.80447 (PMC10406432; doi:10.7554/eLife.80447)
Supplement: Figure 6—figure supplement 1—source data 1. [file elife-80447-fig6-figsupp1-data1.zip › Figure 6- figure supplement 1/Figure 6- figure supplement 1A.pptx]

## Slide 1
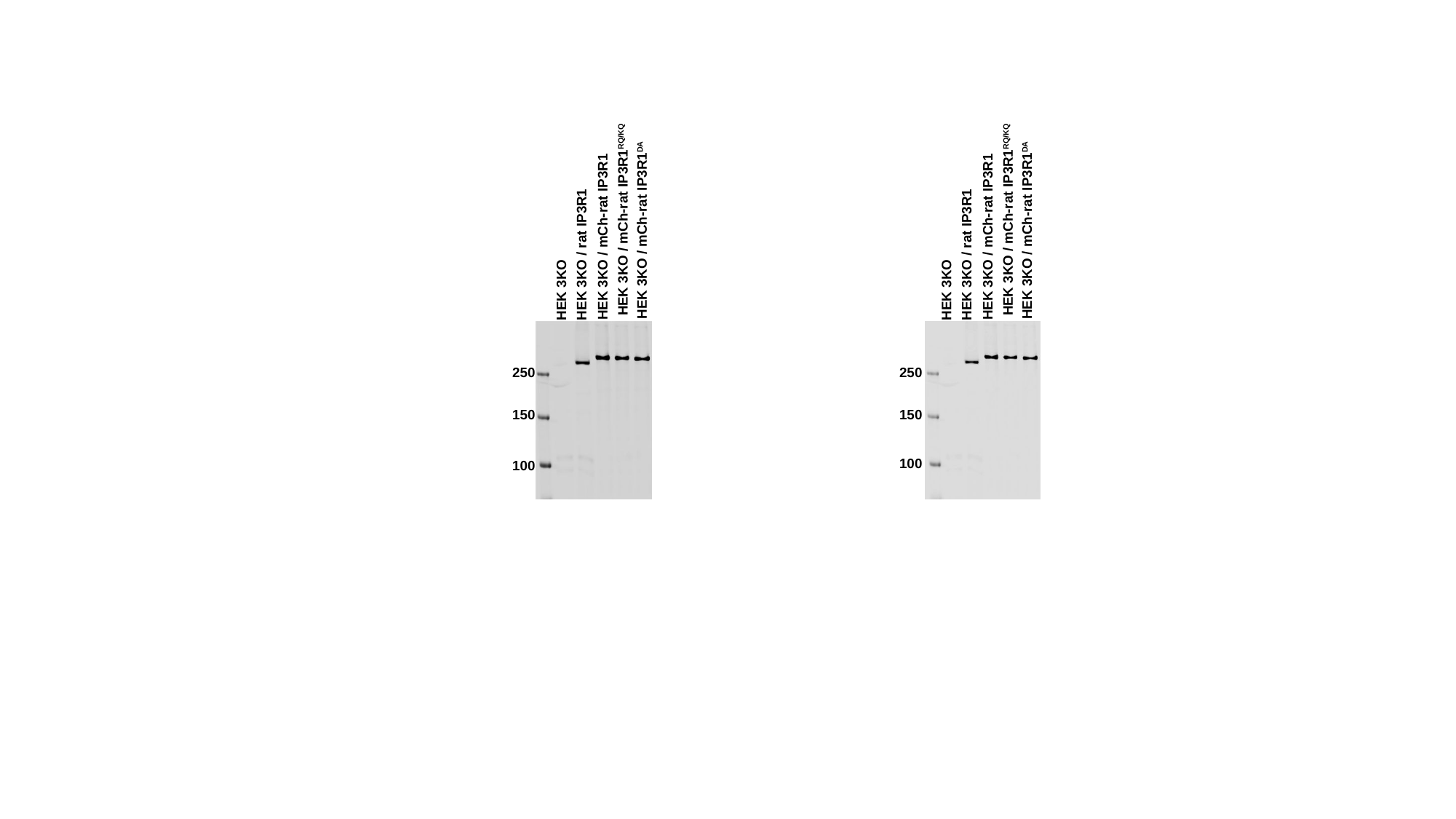

HEK 3KO / mCh-rat IP3R1RQ/KQ
HEK 3KO / mCh-rat IP3R1DA
HEK 3KO / mCh-rat IP3R1
HEK 3KO / rat IP3R1
HEK 3KO
250
150
100
HEK 3KO / mCh-rat IP3R1RQ/KQ
HEK 3KO / mCh-rat IP3R1DA
HEK 3KO / mCh-rat IP3R1
HEK 3KO / rat IP3R1
HEK 3KO
250
150
100
